# A transparent artificial intelligence framework to assess lung disease in pulmonary hypertension

**DOI:** 10.1038/s41598-023-30503-4

**Published:** 2023-03-07

**Authors:** Michail Mamalakis, Krit Dwivedi, Michael Sharkey, Samer Alabed, David Kiely, Andrew J. Swift

**Affiliations:** 1 Department of Infection, Immunity and Cardiovascular Disease, University of Sheffield, Beech Hill Rd, Sheffield, S10 2RX UK; 2 Department of Computer Science, University of Sheffield, 211 Portobello, Sheffield, S1 4DP UK; 3 Department of Cardiology, University of Sheffield, Sheffield Teaching Hospitals Sheffield, Sheffield, S5 7AU UK; 4 Insigneo Institute for in silico Medicine, University of Sheffield, The Pam Liversidge Building, Sheffield, S1 3JD UK

**Keywords:** Computer science, Machine learning, Image processing

## Abstract

Recent studies have recognized the importance of characterizing the extent of lung disease in pulmonary hypertension patients by using Computed Tomography. The trustworthiness of an artificial intelligence system is linked with the depth of the evaluation in functional, operational, usability, safety and validation dimensions. The safety and validation of an artificial tool is linked to the uncertainty estimation of the model’s prediction. On the other hand, the functionality, operation and usability can be achieved by explainable deep learning approaches which can verify the learning patterns and use of the network from a generalized point of view. We developed an artificial intelligence framework to map the 3D anatomical models of patients with lung disease in pulmonary hypertension. To verify the trustworthiness of the framework we studied the uncertainty estimation of the network’s prediction, and we explained the learning patterns of the network. Therefore, a new generalized technique combining local explainable and interpretable dimensionality reduction approaches (PCA-GradCam, PCA-Shape) was developed. Our open-source software framework was evaluated in unbiased validation datasets achieving accurate, robust and generalized results.

## Introduction

Pulmonary hypertension (PH) is a complex condition characterized by elevated pulmonary arterial pressures and presenting with a varying degree of lung parenchymal disease. Computation Tomography (CT) imaging is the gold-standard imaging modality for non-invasive assessment of lung disease and is recommended by the latest European Respiratory Society/European Society of Cardiology PH guidelines^1^. Recent work in the medical literature has highlighted the need to better characterize and quantify lung disease in pulmonary hypertension^2,3^. There is a prognostic significance of lung parenchymal disease on CT with presence of emphysema and ground glass predictive of early mortality^4^.

Deep learning approaches are used to quantify pulmonary ground-glass opacity nodules detection^5^, and emphysema regions using High-Resolution Computed Tomography scans of patients with chronic obstructive pulmonary disease^6^. Moreover, deep learning tries to automate the detection of PH existence or absence^7,8^ and predict elevated pulmonary artery pressure^9^. The current trend is the use of patch-based approaches for texture extraction and feature classification to either segment or classify medical pathologies and regions of interest in a variety of different organs^10–12^. For instance, Tang et al.^13^ proposed a patch-based network with random spatial initialization and statistical fusion on overlapping regions of interest, for three-dimensional abdominal organ segmentation on high-resolution computed tomography. Ben naceu et al.^10^ utilized a deep learning-based selective attention using overlapping patches and multi-class weighted cross-entropy to segment fully automatically a brain tumour. Borne et al.^14^ developed an automatic labelling of cortical sulci using patch and CNN-based segmentation techniques combined with bottom-up geometric constraints. Lastly, Aswathy et al.^15^ used a Cascaded 3D U-net architecture for segmenting COVID-19 infections from lung CT volume images.

Artificial intelligence (AI) approaches show great promise in clinical application particularly in their ability to automatically quantify different radiological lung disease features^16^. However, the use of AI in clinical applications always gives rise to the limitation of introducing bias, and the limitations posed by privacy and security constraints, and lack of transparency and explainability of the networks^17,18^. Translating AI networks from the prototyping version to support clinical stakeholders during routine care brings challenges, especially as decisions impact human lives. It has been observed that when experts interact with AI frameworks, they became biased to reach decisions, and they may be disproportionately inclined to follow the AI’s predictions^19^. That may be problematic because of the AI’s lack of generalization and confidence prediction combine with the risk of learning wrong patterns during the training process. These circumstances motivate the need for transparent AI systems^19^. The latest review of Ciecierski-Holme et al.^20^ highlights that the main limitations of the existing AI studies are related with the lack of successful development and adaptation of well-performing AI tools, the limited available data, the lack of transparent and cost-effective AI tools in low-income and middle-income countries. Shad et al.^21^ state that studies of explainability, uncertainty and bias should be core components of any clinical AI tool studies. Even though there are studies using explainability techniques to increase the transparency of their AI tools they lack generalized approaches as they mainly use local explainable techniques like salience maps, GradCam, or feature engineering approaches^22–24^.

To this end, we developed a transparent AI considering the lack of prediction in high uncertainty circumstances and validating the usability of the system by verifying the correct patterns of learning during the training process. We estimated the epistemic and aleatoric uncertainty of the framework and we developed a new generalized local explainable and interpretable dimension reduction technique (PCA-GradCam, PCA-Shape) to study and validate the prediction of the AI framework. Moreover, we comprehensively studied a pulmonary hypertension multi-classification task by using different deep learning networks (Vgg-16, ResNet-50, DenseNet-121, DenRes-131). We used the deep learning classifier to develop the pathological ratios of lung diseases and to map the 3D anatomical lung models of patients with evidence of pulmonary hypertension. To the authors knowledge, this is the first study to develop a transparent artificial intelligence framework to map and diagnose a patient’s pulmonary hypertension profile in three dimensions.

## Results

We evaluated the results of the multi-classification pulmonary hypertension task in the ‘seen’ validation and test datasets. Moreover, we implemented an ablation study of the framework for different 3D patch sizes, to observe how the variety of the patch sizes influence the performance of the AI framework. Lastly, we validated the AI framework in the ‘unseen’ dataset which includes patients with a challenging pathological pulmonary hypertension profile.

### Multi-classification task in the ‘seen’ validation and testing cohort

**Figure 1:**
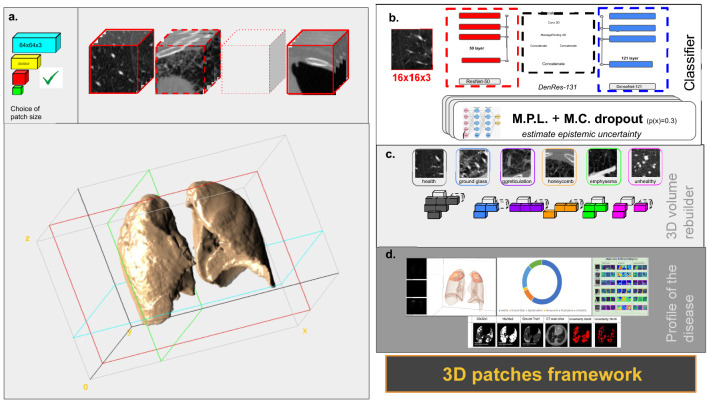
The artificial intelligence framework to diagnose pulmonary hypertension. (**a**) The extraction of 3D patches pipeline. We used four different size of 3D patches to discrete the lungs region, $64 \times 64 \times 3$, $32 \times 32 \times 3$, $16 \times 16 \times 3$, and $8 \times 8 \times 3$. (**b**) Trained deep learning classifier is used to classify each 3D patch in one of the six classes (healthy, ground-glass, ground glass reticulation, honeycomb, emphysema or unhealthy). (**c**) The 3D volume rebuild of the lungs anatomy. (**d**) Profile of the disease of a specific patient. The profile includes the portion of the diseases appeared in the patient’s profile, the 3D anatomical lung model with the diseased areas, the explainability of the deep learning features and the uncertainty estimation of the predictions.

The AUC-ROC curves, precision, recall, and f1-score metrics have been used to evaluate the generalization and accuracy of the networks’ classification. Supplementary Fig. S2 presents the AUC-ROC curves of different deep learning models (DenseNet-121 and DenRes-131) on the datasets. The accuracy of the deep learning classifiers has been tested for a variety of different 3D patch sizes ($64\times 64\times 3$, $32\times 32\times 3$, $16\times 16\times 3$, and $8\times 8\times 3$, Supplementary Fig. S2a–d, e–h, i–l and m–p, from left to right, respectively). The performance of the networks decreased as we reduced the 3D patches size of the multi-classification task. The best results scored by the $64\times 64\times 3$ patch size, with an AUC-ROC higher than 98.0% in DenseNet-121 and higher than 96.8% in the DenRes-131 in the validation cohort for all the different classes. In the test cohort (Supplementary Fig. S2i–p) DenseNet-121 performed higher than 96.1% AUC-ROC accuracy in all the classes, whilst DenRes-131 scored higher than 90.9%. The $32\times 32\times 3$ patch size in the validation cohort DenseNet-121 outperformed the accuracy of DenRes-131 in honeycomb, emphysema, and abnormal classes and it was outperformed by the accuracy of DenRes-131 in normal, pure ground glass, and ground glass reticulation classes. On the other hand, in the test cohort DenRes-131 outperformed the accuracy of the DenseNet-121 in all classes, verifying the higher generalization of the model compared to the DenseNet-121 (Fig. 1).

**Figure 2:**
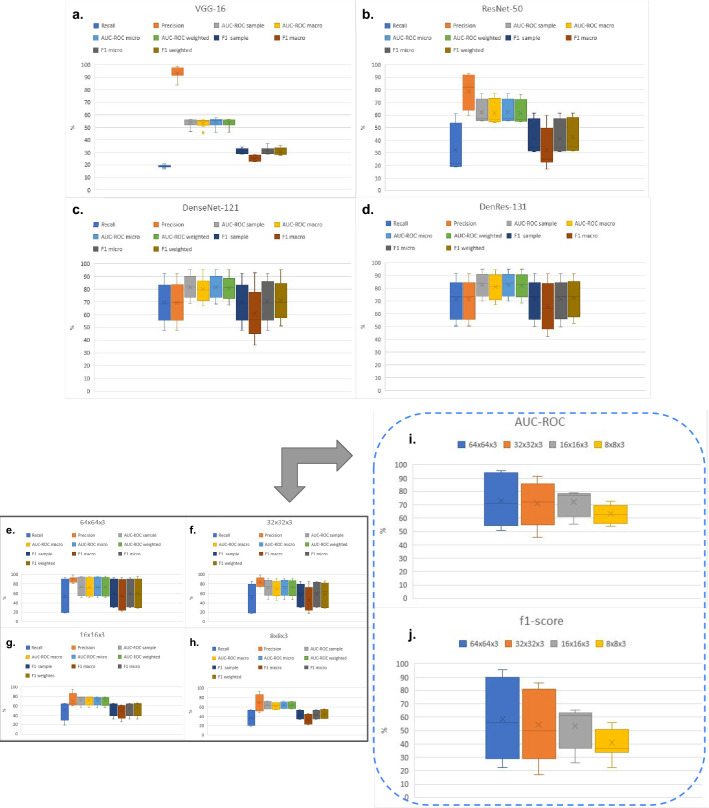
Box and Whisker plots results of Vgg-16, ResNet-50, DenseNet-121, and DenRes-131 for the multi-classification task. (**a**–**d**) Box-plots results for the different deep learning networks (Vgg-16, ResNet-50,DenseNet-121, and DenRes-131, respectively) for the combine results of all the different size of 3D patch sizes. The results presented are a variation of metrics (Recall, Precision, AUC-ROC, and f1-score) scores. (**e**–**h**) Box-plots results for the different patch sizes ($64 \times 64 \times 3$, $32 \times 32 \times 3$, $16 \times 16 \times 3$, and $8 \times 8 \times 3$ height, width and depth respectively) of all the deep learning networks combine results. (**e**–**h**) are presented the results of a variation of metrics (Recall, Precision, AUC-ROC, and f1-score). (**i**,**j**) Summarizing the f1-score and AUC-ROC metrics results.

Figure 2 presents different metric scores (f1-score, AUC-ROC, Recall, Precision) of the deep learning networks (VGG-16, ResNet-50, DenseNet-121 and DenRes-131) for the test cohort of the ‘seen’ dataset. Figure 2a–d, shows that DenseNet-121 and DenRes-131 outperformed ResNet-50 and VGG-16 in all the metrics except precision. DenRes-131 delivered the best results in all metrics compared with DenseNet-121. Figure 2e–h, present the results of the different patch sizes. Figure 2e–h highlights all the metrics scores for each patch size for DenRes-131 and Fig. 2i,j, we summarize the AUC-ROC and f1-score values of the four different patch sizes. The most robust results were scored for the $16\times 16\times 3$ and $8\times 8\times 3$ sizes followed by the $32\times 32\times 3$ and $64\times 64\times 3$. The highest average value was for the $16\times 16\times 3$ followed by the $32\times 32\times 3$ and $64\times 64\times 3$ in the AUC-ROC and f1-score metrics, respectively. Summarizing, in the multi-classification task the highest performance was by the DenRes-131 network and $16\times 16\times 3$ patch size followed by the $32\times 32\times 3$ patch size.

### Validation of the AI framework in the ‘unseen’ cohort

**Figure 3:**
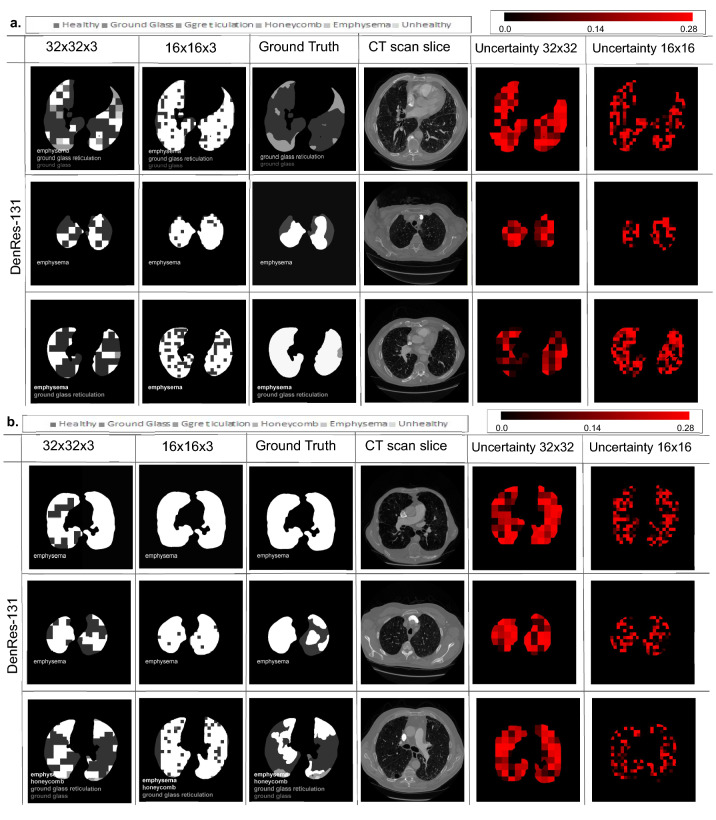
The multi-classification results of six different patients of the ‘unseen’ cohort (PART 1). (**a**,**b**) From left to right the prediction results of the patch sizes ($32 \times 32 \times 3$, and $16 \times 16 \times 3$), the radiologist ground truth, the patient CT slice of short axis, the uncertainty mapping of $32 \times 32 \times 3$, and the uncertainty mapping of $16 \times 16 \times 3$. The six different classes are showing in gray-scale colours. The uncertainty scale is with red-scale from 0.00 to 0.30 probability.

**Figure 4:**
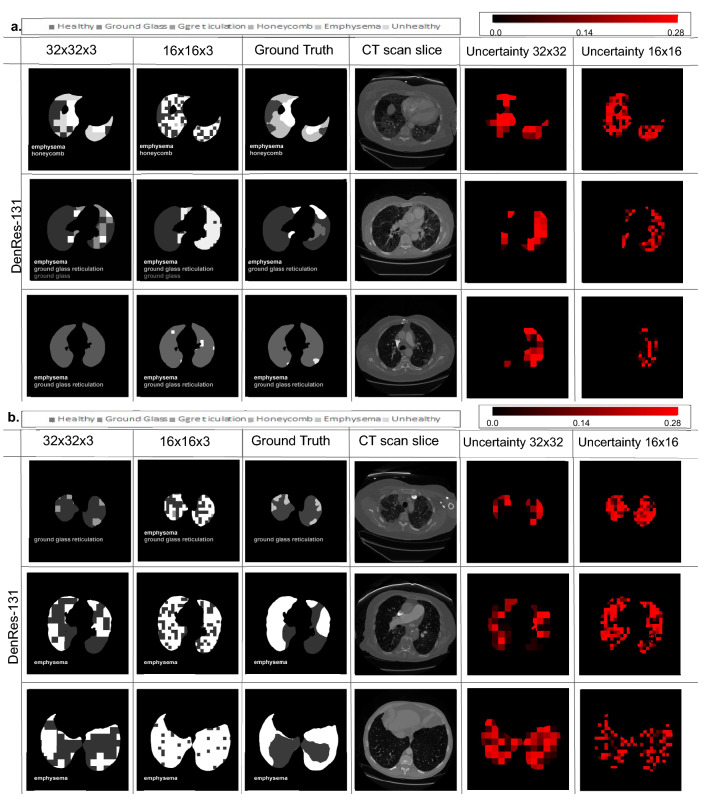
The multi-classification results of six different patients of the ‘unseen’ cohort (PART 2). (**a**,**b**) From left to right the prediction results of the patch sizes ($32 \times 32 \times 3$, and $16 \times 16 \times 3$), the radiologist ground truth, the patient CT slice of short axis, the uncertainty mapping of $32 \times 32 \times 3$, and the uncertainty mapping of $16 \times 16 \times 3$. The six different classes are showing in gray-scale colours. The uncertainty scale is with red-scale from 0.00 to 0.30 probability.

To evaluate the 3D-patch framework in the ‘unseen’ cohort we used the measurements of Jaccard score, Hamming distances, Root Mean Square Error (RMSE), f1-score, recall, precision, Matthews correlation coefficient (MCC), and accuracy. Table 1 presents the scores of ResNet-50, DenseNet-121 and DenRes-131 for the four different patch sizes in the full lungs slices of the ‘unseen’ cohort. In $64\times 64\times 3$ the ResNet-50 outperformed the other networks with 74.63% Jaccard score and 1.246 RMSE score. In $32\times 32\times 3$ and $16\times 16\times 3$ DenRes-131 outperformed the other networks with 91.83% Jaccard score, 5.96 mm Hamming distances and 0.855 RMSE score and 89.01% Jaccard score, 7.99 mm Hamming distances and 1.015 RMSE score respectively. DenseNet-121 outperformed all the networks in $8\times 8\times 3$ patch size with 69.20% Jaccard score, 18.27 mm Hamming distances and 1.361 RMSE score. The best performance of the networks was in $32\times 32\times 3$ patch sizes followed by $16\times 16\times 3$. Generally, the highest average score was from the DenRes-131 and the $32\times 32\times 3$ patch size (91.83%, 5.96 mm, 0.855, 93.87%, 93.42%, 96.54%, 93.69% and 80.21%, respectively). The most robust results (lowest standard deviation) were presented in the DenRes-131 and $16\times 16\times 3$ patch size for the RMSE, f1-score, precision, accuracy and MCC in the $16\times 16\times 3$ (0.32, 4.87%, 2.20%, 3.27%, and 7.21%, respectively), as mentioned in Fig. 2. The results of DenRes-131 for the $32\times 32\times 3$ and $16\times 16\times 3$ patch sizes in the ‘unseen’ cohort are highlighted in Figs. 3 and 4. The figures presents twelve different patients of the ‘unseen’ cohort ( six in PART 1 Fig. 3 and six in PART 2 Fig. 4) by using DenRes-131 with $32 \times 32 \times 3$ and $16 \times 16 \times 3$ patch sizes. From left to right the predicted results of the patch sizes ($32 \times 32 \times 3$, and $16 \times 16 \times 3$), the radiologist ground truth, the patient CT slice of short axis, the uncertainty mapping of $32 \times 32 \times 3$, and the uncertainty mapping of $16 \times 16 \times 3$ are presented. The six different classes are shown in grey-scale colours. The uncertainty scale is with red scale from 0.00 to 0.30 probability. To compare the performance of each patch size we presented the ground truth based on the two expert radiologists and the correspondence CT slice of the patient. In most cases the $32\times 32\times 3$ estimated better than $16\times 16\times 3$, except for the 3rd, 2nd and 1st cases of Figs. 3a, 4a,b respectively. The $32\times 32\times 3$ patch size delivered clinically appropriate level of prediction, contrary to $16\times 16\times 3$ which in most of the cases overestimated the results. The framework’s predictions and robustness were strengthened by the uncertainty estimation mapping of each prediction probability. Figures 3 and 4 presented the uncertainty mapping of the prediction.The combination of uncertainty prediction and the probability prediction strengthens the trustworthiness of the AI tool, as for high uncertainty experts can ignore the prediction.

**Table 1:** Quantitative evaluation metrics of the AI framework on the unseen dataset.

Metric	ResNet-50	DenseNet121	DenRes-131
The AI framework for the $64\times 64\times 3$ patch size			
Jaccard score (%)	74.63 ± 17.95	70.12 ± 18.32	69.21 ± 17.89
Hamming distances (mm)	16.21 ± 6.21	18.01 ± 8.01	18.07 ± 9.03
Root mean square error	1.246 ± 0.510	1.282 ± 0.634	1.281 ± 0.700
f1 score (%)	77.33 ± 10.02	73.21 ± 13.54	73.01 ± 13.77
Recall score (%)	78.21 ± 9.87	74.01 ± 14.01	73.99 ± 14.04
Precision score (%)	78.33 ± 9.02	74.32 ± 14.21	74.35 ± 14.98
Accuracy (%)	78.23 ± 10.00	74.00 ± 15.01	73.12 ± 16.00
MCC (%)	67.23 ± 17.21	64.12 ± 18.12	63.89 ± 19.00
The AI framework for the $32\times 32\times 3$ patch size			
Jaccard score (%)	69.41 ± 21.94	90.53 ± 4.38	*91.83 ± *3.48
Hamming distances (mm)	17.29 ± 7.29	6.34 ± 3.82	*5.96 ± *3.17
Root mean square error	1.171 ± 0.419	0.904 ± 0.54	*0.855 ± 0.40
f1 score (%)	71.23 ± 10.11	92.06 ± 5.40	*93.87 ± 4.20
Recall score (%)	70.12 ± 10.33	93.21 ± 4.11	*93.42 ± *2.84
Precision score (%)	71.22 ± 10.43	94.53 ± 2.52	*96.54 ± 2.82
Accuracy (%)	70.15 ± 10.32	93.02 ± 3.93	*93.69 ± 3.90
MCC (%)	65.34 ± 20.32	77.74 ± 8.46	*80.21 ± 7.83
The AI framework for the $16\times 16\times 3$ patch size			
Jaccard score (%)	70.05 ± 20.67	87.70 ± 8.90	89.01 ± 5.81
Hamming distances (mm)	17.13 ± 7.11	8.08 ± 5.26	7.99 ± 4.48
Root mean square error	1.146 ± 0.409	1.035 ± 0.42	1.015 ± *0.32
f1 score (%)	72.01 ± 10.00	92.06 ± 5.10	92.27 ± *4.87
Recall score (%)	71.00 ± 10.01	90.67 ± 4.53	91.64 ± 4.33
Precision score (%)	71.87 ± 10.67	95.71 ± 2.08	95.65 ± *2.20
Accuracy (%)	70.78 ± 10.78	90.88 ± 4.74	91.51 ± *3.27
MCC (%)	66.01 ± 20.01	73.10 ± 11.71	74.80 ± *7.21
The AI framework for the $8\times 8\times 3$ patch size			
Jaccard score (%)	68.56 ± 21.09	69.20 ± 18.52	69.10 ± 18.20
Hamming distances (mm)	16.61 ± 6.23	18.27 ± 9.91	18.31 ± 9.83
Root mean square error	1.587 ± 0.355	1.361 ± 0.839	1.432 ± 0.840
f1 score (%)	74.13 ± 13.12	72.51 ± 15.54	72.41 ± 15.74
Recall score (%)	72.41 ± 10.82	73.41 ± 16.01	73.49 ± 16.04
Precision score (%)	72.63 ± 12.21	74.32 ± 17.22	73.35 ± 16.98
Accuracy (%)	73.53 ± 11.90	73.60 ± 17.11	73.10 ± 16.89
MCC (%)	61.43 ± 18.20	64.02 ± 19.18	63.59 ± 19.20

$*$The highest performance of each metric score.

Significant values are given in bold.

**Figure 5:**
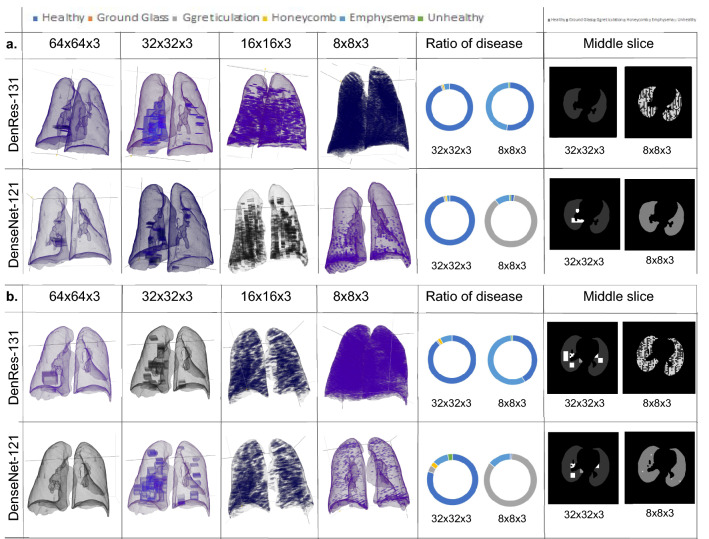
Results of two different patient of the ‘seen’ testing cohort. (**a**,**b**) The 3D mapping of two patients (**a**,**b**) for the DenseNet-121 and DenRes-131 in the multi-classification task. Four different 3D patch sizes volume: $64 \times 64 \times 3$, $32 \times 32 \times 3$, $16 \times 16 \times 3$, and $8 \times 8 \times 3$ height, width and depth respectively are presented. From left to right the four different 3D anatomical lung models, the ratio of diseases results of the patch sizes $32 \times 32 \times 3$, and $8 \times 8 \times 3$, and the prediction results of the middle slice of short axis.

Figure 5a,b shows the 3D anatomical lung models with the diseased lung areas for two patients in the seen testing cohort. Moreover, Fig. 5a,b presents the ratio of the diseases and the middle slice results of the deep learning networks (DenseNet-121 and DenRes-131) for the four different patch sizes. The network with most accurate results based on Table 1, Figs. 3 and 4 was the DenRes-131 for the $32\times 32\times 3$ patch size. Therefore, $32\times 32\times 3$ patch size and the DenRes-131 network were the most robust and generalisable combination for the multi-classification task. To this end, the 3D-patch framework overestimates the diseases in cases of small patch size ($8\times 8\times 3$) and underestimates in the large patch size ($64\times 64\times 3$).

### Generalized explanation of the AI framework

**Figure 6:**
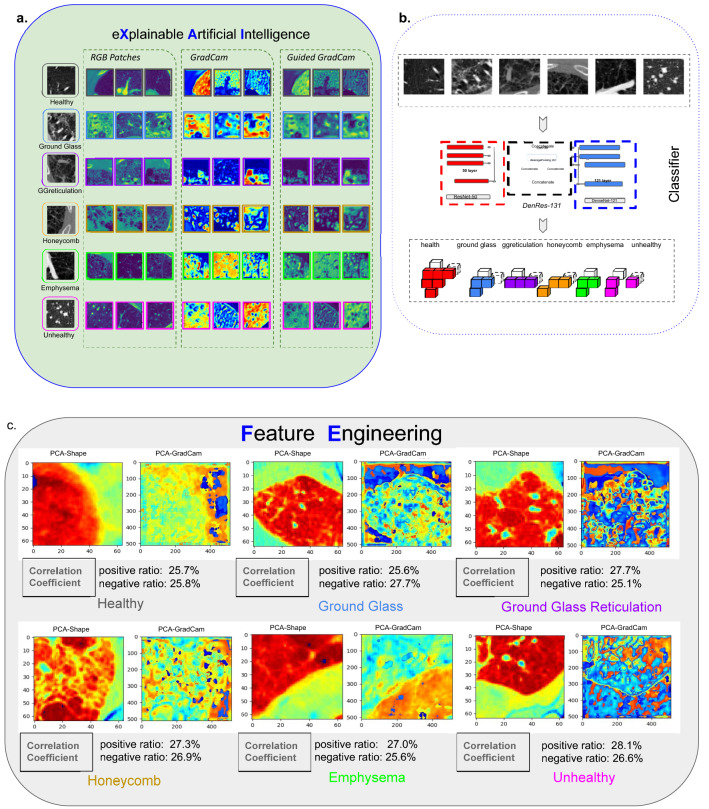
The local explainability results of DenRes-131 and the $32 \times 32 \times 3$ patch size of the multi-classification task. (**a**) The colorful RGB patches, GradCam, and guided GradCam results of the six classes (healthy, ground glass, ground glass reticulation, honeycomb, emphysema and unhealthy). (**b**) the structure of DenRes-131 network. (**c**) The PCA zero component of the patch images (PCA-Shape) and GradCam results (PCA-GradCam) of each class for a four components analysis. The correlation coefficient between the PCA-Shape (Average image) and the PCA-GradCam (PCA GRADCAM) with the negative and positive ratio is presented.

The most accurate deep learning network was the DenRes-131 with the $32\times 32\times 3$ patch size (Fig. 6b). Therefore, we studied the local and generalized explainability of that case.

**Figure 7:**
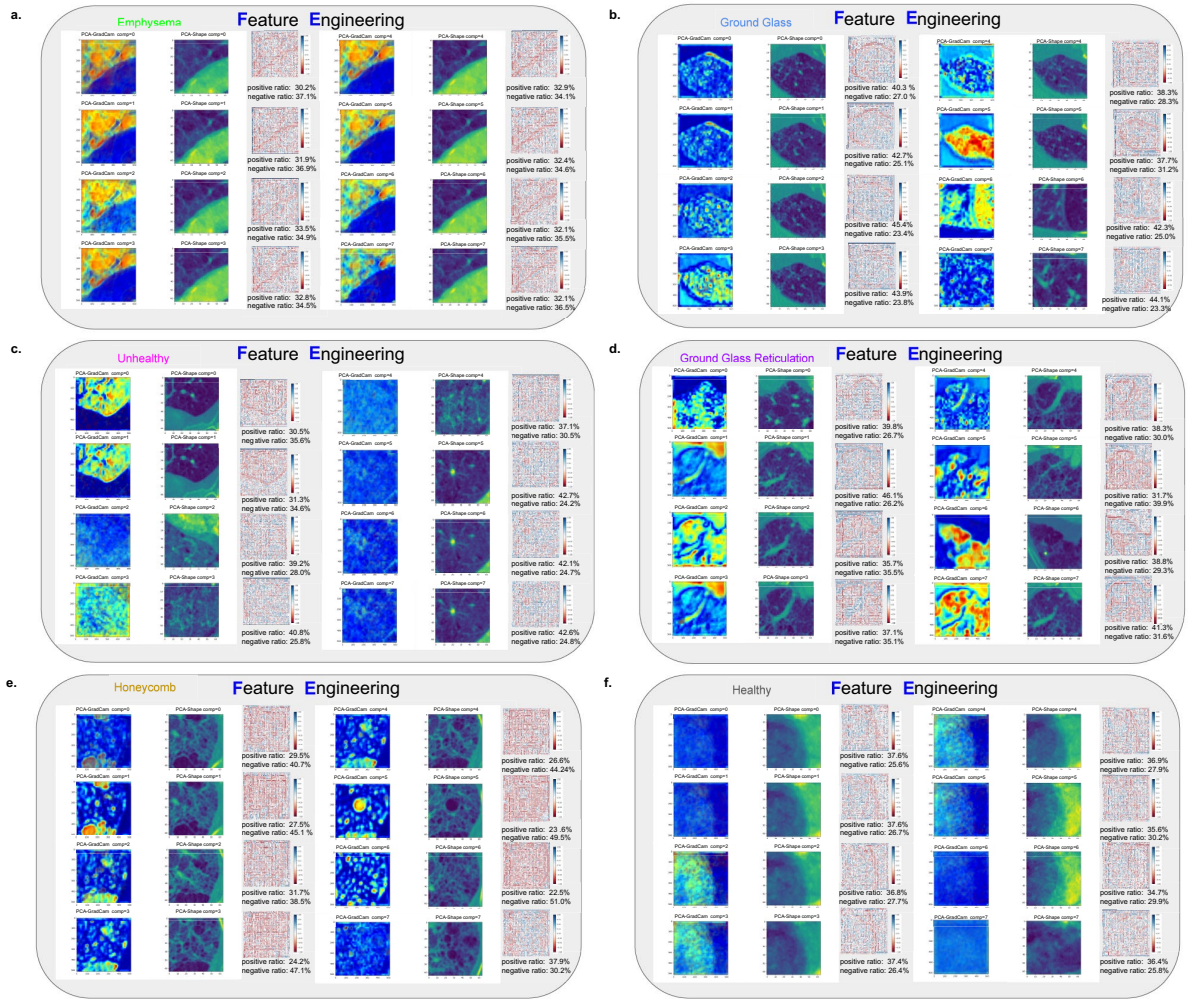
The generalized combined explainable technique of local explanable and interpretable reduced dimensionality techniques (PCA-GradCam, PCA-Shape). (**a**–**f**) The PCA of the patch images (PCA-Shape) and GradCam results (PCA-GradCam) of each class for the eight components analysis. The correlation coefficient of each component’s PCA-Shape and PCA-GradCam with the negative and positive ratio is presented. For each class from left to right, top to bottom the eight components results of the PCA analysis. The results are based on the DenRes-131 and $32 \times 32 \times 3$ patch size for the multi-classification task.

**Figure 8:**
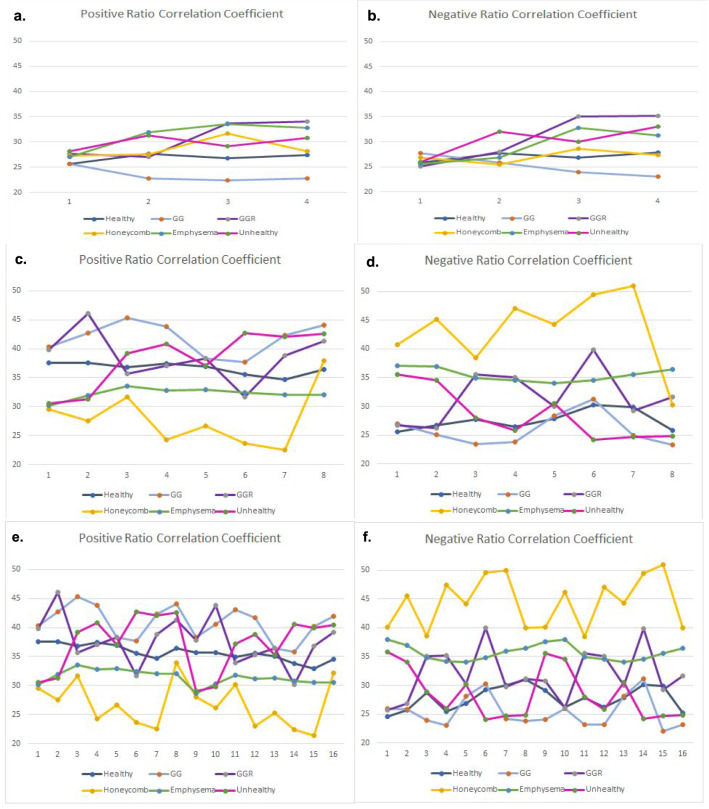
The positive and negative ratio results of each class with respect of the PCA analysis of the four, eight and sixteen components respectively. (**a**,**b**) The positive (**a**) and negative (**b**) ratio results of each class with respect of the PCA analysis of the four components. The results are based on the DenRes-131 and $32 \times 32 \times 3$ patch size for the multi-classification task (**c**–**f**) The positive and negative ratio results of each class with respect of the PCA analysis of the eight (**c**,**d**) and sixteen components (**e**,**f**) respectively. The results are based on the DenRes-131 and $32 \times 32 \times 3$ patch size for the multi-classification task.

Figure 6a shows the local explainable results of DenRes-131 for the six-classification task for the $32 \times 32 \times 3$ patch size. The local explanation is a collection of the colourful RGB patches, GradCam, and guided GradCam results of the six classes (healthy, ground glass, ground glass reticulation, honeycomb, emphysema and unhealthy, Fig. 6a). The GradCam and guided GradCam was extracted from the convolutional layer after the concatenation of the ResNet-50 and DenseNet-121 networks. Regarding the guided GradCam results of all the six classes, the networks were focusing on the correct area of interest (healthy and pathological lung area). However, the evaluation of these three samples was highly biased to conclude about the general correct learning patterns of the network as GradCam is a local explainable method. To this end, we developed a combination technique that utilized the PCA of different components (4,8 and 16) in the total sample of the patch images (PCA-Shape) and their corresponding local GradCam images (PCA-GradCam) to evaluate the learning patterns of each class. Figure 6c shows the PCA zero component of the PCA-Shape and the PCA-GradCam results of each class for the four principal component analysis. Moreover, the correlation coefficient of the PCA-Shape and the PCA-GradCam with the total negative and positive pixels ratio is presented. The results showed that the network focused on the correct learning patterns (positive ratio higher than negative) in honeycomb, emphysema and unhealthy classes. On the other hand, the network learned wrong patterns in the ground glass class. The healthy class had almost the same number of the negative and positive ratio between the zero PCA component of PCA-Shape and PCA-GradCam. Figure 8a,b presents the positive and negative ratio results of each class with respect to the PCA analysis of the four components. Even if the zero-component showed that the network learned the patterns of the unhealthy and ground glass reticulation classes corectly, the other three components showed that the network did not, as they had higher negative pixels ratio values than positive pixels ratio values (Fig. 8a,b). This instability between the components of the PCA analysis (4 components) justified the need to study different numbers of PCA components (8, and 16) to conclude about the most stable dimension reduction analysis to generalize the local explainable observations. Therefore we present the eight components of the PCA-Shape and PCA-GradCam analysis for each class (Fig. 7). Figure 7a–f shows the generalized explainable results of DenRes-131 for the $32 \times 32 \times 3$ patch size of multi-classification task. We computed the correlation coefficient of the PCA-Shape and the PCA-GradCam. We further computed the negative and positive pixels ratio of the correlation between the PCA-Shape and PCA-GradCam, to evaluate the generalized correct learning pattern of the network in each class.

The correlation coefficient of positive and negative pixels ratio is a way to evaluate the false positive or negative pixels and the true positive or negative pixels of the network’s learning patterns (analytical explanation of the network’s learning pattern of the generalized technique in: Supplementary material subsection 2.4). For instance, in the healthy and unhealthy classes the component zero, one and four of the PCA, focused correctly in the lung area. Moreover, the network correctly learned the pattern in the component six and seven of the ground glass reticulation class. In the ground glass the network was focusing correctly in the lung area of interest in the components zero, one, and three. However, the network focused additionally in the peripheral areas that increase the false positive and false negative pixels ratio, and this concludes as a wrong learning pattern in total for the classes of interest. As the PCA-Shape images had high intensity pixels in lung areas that are not of interest and low intensity pixels in lungs areas that are of interest in the majority of the eight components, the network correctly learned the patterns when the positive correlation coefficient ratio was lower than the negative correlation coefficient pixels ratio. The healthy, ground glass reticulation, ground glass and unhealthy classes had higher positive pixels ratio than negative pixels ratio in all the eight components. On the other hand, emphysema, honeycomb had lower positive pixels ratio than negative pixels ratio in all the eight components. To this end, the network correctly learned the patterns of emphysema, honeycomb and unhealthy classes but learned the other three classes wrongly.

Figure 8a–f presents the positive and negative pixels ratio results of each class with respect to the PCA analysis with four, eight and sixteen components. This figure summarizes the observations we discussed above. The PCA with eight components (Fig. 8c,d) has the same behaviour as the PCA with sixteen components (Fig. 8e,f). Therefore, the results of PCA analysis with eight components are more trusted compared with the PCA analysis of four components (Fig. 8a,b).

### Uncertainty estimation

**Figure 9:**
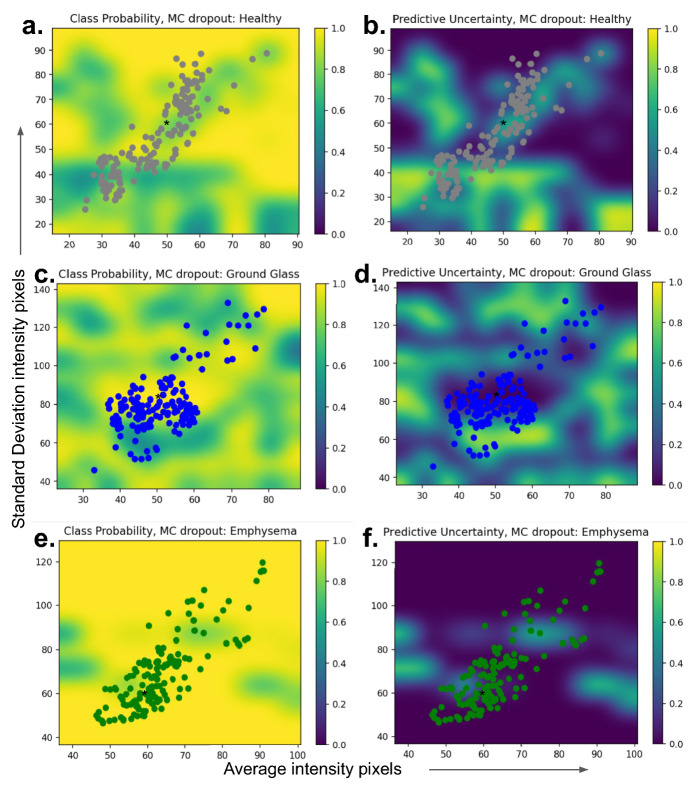

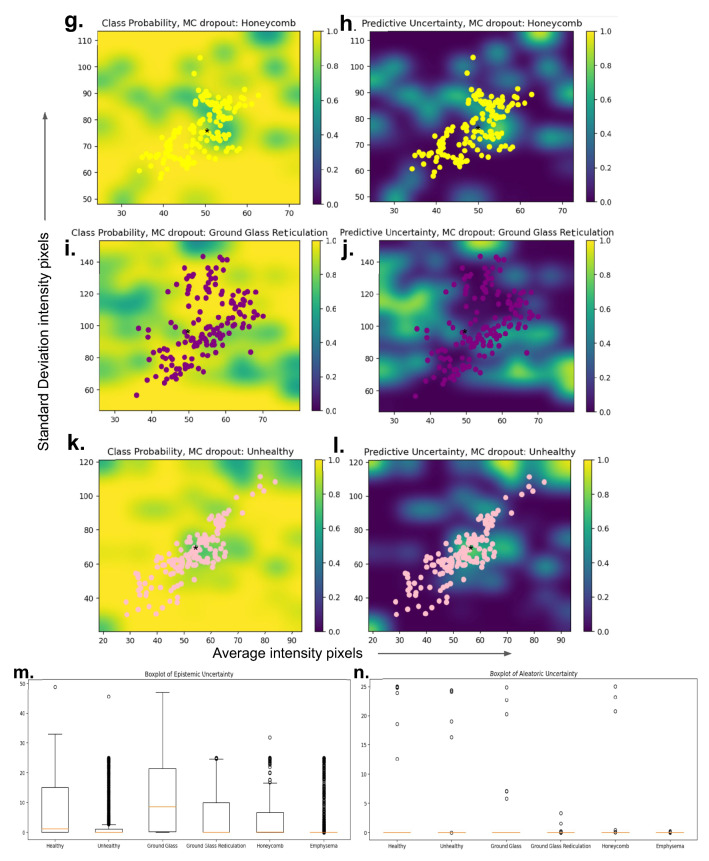
The epistemic and aleatoric uncertainty profile of the ‘seen’ testing dataset of each class for the DenRes-131 deep learning network. (**a**–**l**) The normalized class probability and predictive uncertainty (epistemic uncertainty) of each class. Each patch image class presented by a different colour circle (patch size of $32\times 32\times 3$) with respect of the average and standard deviation of the intensity pixels. (**m**,**n**) the box and Whisker plots results of the epistemic (**m**) and aleatoric (**n**) uncertainty for each class.

A crucial dimension for a transparent artificial intelligence framework is the uncertainty estimation. Figure 9 shows the aleatoric and epistemic uncertainty of the testing internal cohort and the DenRes-131 network, respectively. Figure 9m,n shows the boxplots of the epistemic and aleatoric uncertainty for each class. The healthy, unhealthy, ground glass, and honeycomb classes had a high value of aleatoric uncertainty. The emphysema and ground glass reticulation classes had a low value of aleatoric uncertainty. On the other hand, the ground glass, and healthy classes followed by the ground glass reticulation and honeycomb classes had high epistemic uncertainty. Figure 9a–l presents the normalized class probability and predictive uncertainty (epistemic) of each class. The coloured circles are the patch images (size of $32\times 32\times 3$) of each class with respect to the average and standard deviation of the intensity pixels. The emphysema was the most robust prediction class (high class probability, low predictive uncertainty) followed by the honeycomb classes. The ground glass was the least robust prediction class followed by the healthy and ground glass reticulation.

### Statistical analysis results

We utilized ANOVA analysis with $\text {p}$-value 0.05 to calculate the statistically significant differences between the different deep learning classifiers. All the results of the models were statistically significantly different with $\text {{p}}<0.05$. The DenRes-131 had significantly different results compare with the DenseNet-121 with a $\text {{p}}$-value of 0.04.

## Discussion

Translating AI networks from the prototyping version to support clinical stakeholders during routine care is highly dependent on the trustworthiness of the AI tool. In most of the existent AI studies there is a lack of successful development and adaptation of well-performing and context-specific AI tools. Even though there are studies using explainability techniques to increase the transparency of the AI tools, they lack generalization as they mainly use local explainable techniques. A transparent AI tool needs to include dimensions like explainability, uncertainty and bias for any clinical application as part of its core study. To this end, in this study we developed and analysed a transparent artificial intelligence framework to map the 3D anatomical models of patients with evidence of lung diseases in pulmonary hypertension.

To be sure about how thoroughly the framework evaluates in functional, operational, and usability dimensions we studied the framework’s performance in different patch sizes ($64\times 64\times 3$, $32\times 32\times 3$, $16\times 16\times 3$, $8\times 8\times 3$) in a multi-classification task and we trained and tested different established deep learning networks. The framework was evaluated by an unbiased validation profile of internal ‘seen’ and ‘unseen’ multi-scan and multi-vendors cohorts. The results highlighted that the patch size of $32\times 32\times 3$ (and in some cases $16\times 16\times 3$) was the most accurate, robust and generalised. The DenRes-131 network was the most accurate framework followed by the DenseNet-121 in the multi-classification task. We verified the accurate and robust predictions of the framework in the ‘unseen’ cohort achieving metrics scores such as 91.83 ± 3.48% Jaccard score, 5.96 ± 3.17 mm Hamming distances, 80.21 ± 7.83% MCC, 93.69 ± 3.90 accuracy, and 0.855 ± 0.40 Root Mean Square Error. Lastly, we justified that the framework predicts high performance and robustness in the emphysema and the honeycomb diseases but lacks accurate prediction of the ground glass reticulation and the ground glass diseases.

To verify the transparency and trustworthiness of the AI framework we studied the uncertainty estimation of the network’s prediction, and we tried to explain the generalized learning patterns of the network. Therefore, a new generalized technique combines local explainable and interpretable dimension reduction approaches (PCA-GradCam, PCA-shape) was developed. We studied PCA analysis of different component numbers (4, 8 and 16) and we concluded that the eight components of the PCA-Shape (PCA of total patch images) and the PCA-GradCam (PCA of total GradCam images) analysis were the most robust. Moreover, we computed the correlation coefficient of the PCA-Shape and PCA-GradCam and the negative and positive pixels ratio of the correlation between them. In this way, we evaluated the generalized correct and wrong learning pattern of the network in each class. The correlation coefficient of positive and negative pixels ratio was an approach to evaluate the false positive or negative pixels and the true positive or negative pixels of the network’s learning patterns. The network learned correct patterns in the emphysema and honeycomb classes and wrong patterns in the ground glass and ground glass reticulation classes. By using augmentation techniques with Monte Carlo simulations and Monte Carlo dropout layers we estimated the aleatoric and epistemic uncertainty of each class. The dataset had high aleatoric uncertainty in the ground glass, unhealthy, healthy and honeycomb diseases and the framework predicts high epistemic uncertainty in the ground glass, ground glass reticulation and healthy diseases.

Even if we delivered a transparent AI framework there is a limitation about the performance of the AI in the classes with high uncertainty like the ground glass and healthy classes. We can solve this problem by applying different pre-processing techniques, by increasing the samples variability of the high uncertainty classes and by applying domain adaptation techniques to increase the AI framework performance in the out of the distribution samples. Therefore as future work we will increase the variability of the datasets including more clear cases of ground glass and ground glass reticulation patients to reduce the aleatoric uncertainty. Furthermore, we will apply domain adaptation techniques in the classifiers like few shots, to increase the accuracy of the prediction and decrease the uncertainty in the ground glass and ground glass reticulation classes. Lastly, we aim to create a thresholding validation protocol to identify the appropriate threshold difference between the positive and negative pixels ratio of the correlation coefficient method of our combined local explainable and global interpretable techniques to justify uniquely and unbiasedly the correct and wrong learning patterns of the networks.

Our AI framework was tested in an unbiased validation protocol which accurately captures ordinary clinical trials, and it delivered accurate robust and generalized performance with uncertainty prediction probabilities and generalized explanations (Fig. 1d).

## Methods

### Dataset protocol

The dataset protocol and methods were performed in accordance with relevant guidelines and regulations and approved by ASPIRE registry (Assessing the Spectrum of Pulmonary Hypertension Identified at a Referral Centre), reference c06/Q2308/8; REC 17/YH/0016. This study complies with the Declaration of Helsinki. We confirm that all experiments were performed in accordance with relevant guidelines and regulations. Informed consent was obtained from all subjects and/or their legal guardian(s).

#### Validation datasets protocol

To train and evaluate the networks in the multi-classification task, we used a cohort of 84 patients (‘seen’ cohort). From the ‘seen’ cohort 75 patients were used for training/validation and 9 for testing. As the multi-classification task was based on a patch oriented deep learning approach, we utilized four different patch sizes ($64\times 64\times 3$, $32\times 32\times 3$, $16\times 16\times 3$ and $8\times 8\times 4$) to study the sensitivity of the networks’ predictions. For each patch size we used a balanced number of images in each class, and a 70/30 validation split protocol during training. More analytically, for the training task we utilized for each class: 8000, 40,000, 185,000 and 600,000 images for the $64\times 64\times 3$, $32\times 32\times 3$, $16\times 16\times 3$ and $8\times 8\times 3$ patch sizes, respectively. For the testing task we utilized for each class: 4000, 22,000, 50,000 and 140,000 images for the $64\times 64\times 3$, $32\times 32\times 3$, $16\times 16\times 3$ and $8\times 8\times 4$ patch sizes respectively. To evaluate the AI framework in an out of distribution task (OoD) we used a cohort of 19 patients with full annotated PH diseases (‘unseen’ cohort). This dataset was a collection of patients different from the ‘seen’ dataset with multi-vendors and multi-scans variability.

#### CT imaging protocol

For the ‘seen’ cohort all patients were diagnosed with PH between Feb 2001 and Jan 2019. They were identified in a specialist PH referral centre using the ASPIRE (Assessing the Spectrum of Pulmonary Hypertension Identified at a Referral Centre) registry. Around 17,500 CT slices were divided into six regions: healthy lungs, ground glass, ground glass reticulation, honeycomb, emphysema, unhealthy lungs. These classes were manually labelled in each slice by two specialist radiologists and differences resolved through consensus. The unhealthy class was a combination of lung diseases observations with low frequency in the dataset (centrilobular ground glass, fibrosis, consolidation and low attenuation). We combined these diseases in the ‘unhealthy’ class, as we needed to extract a balance training dataset with same number of samples in each class. We used these regions to train and validate the multi-classification task. The ‘unseen’ cohorts included a collection of 19 patients with a diagnosis of PH from the ASPIRE registry. The unseen cohort was a collection of five anatomical CT slice levels exhaustively labeled by specialist radiologists (KD and AS with 3-years and 10-years experience) for each patient. The anatomical levels chosen were top of the aortic arch, bifurcation of the trachea, main pulmonary artery bifurcation, mitral valve, and diaphragm.

#### Cohort’s pre-processing image analysis

Image analysis techniques have been applied to all slices to reduce the effect of noise and increase the signal-to-noise ratio (SNR), using noise filters such as curvature anisotropic diffusion image filters^25^. Moreover, we normalized the images and we have used data augmentation techniques including rotation (rotation around the center of the image by a random angle in the range of $-15^{\circ }$ to $15^{\circ }$), width shift range (width shift of the image by up to 20 pixels), height shift range (height shift of image by up to 20 pixels), and ZCA whitening (add noise in each image)^26^.

### Modeling framework

We developed a patch-oriented AI framework to map the 3D anatomical models of patients with lung disease in pulmonary hypertension. The framework has four steps: the extraction of the 3D patches, the classification of each patch, the volume rebuild of the 3D anatomical model, and the analysis and evaluation of the pulmonary hypertension profile of the lungs (Fig. 1).

To train the classifiers we extracted volume patches from the initial NIFTI and DICOM files of the CT images. We segmented the lung region using a nn-unet network^27^ and we used a variate of different sizes of the volume patch sizes to evaluate the performance of the classifiers. We have used four different patch sizes of $64 \times 64 \times 3$, $32 \times 32 \times 3$, $16 \times 16 \times 3$, and $8 \times 8 \times 3$ height, width and depth respectively (Fig. 1a). The extracted patches were used for training, validation and testing (‘seen’ cohort) for the deep learning classifiers.

We solved a multi-classification problem of six different classes: healthy, ground-glass, ground glass reticulation, honeycomb, emphysema and unhealthy. To evaluate and optimize the solution of the multi-classification task we used three established networks VGG-16, DenseNet-121 and ResNet-50 and one state-of-the-art deep learning network DenRes-131^28^. VGG-16 is a well-established convolutional neural network (CNNs) with a combination of pooling and convolution layers^29^. ResNet-50 is a deep network, in which all layers have the same number of filters as the number of the output feature size. In case the output feature size is halved, the number of filters is doubled, thus reducing the time complexity per layer^30^. DenseNet-121 is an efficient convolutional network. The network comprises of deep layers, each of which implements a nonlinear transformation. Hauang et al.^31^ introduced a unique connectivity pattern information flow between layers to direct connecting any layer to all subsequent layers. DenRes-131 is a modified version of^28^ network which has two dropout layers to estimate the epistemic uncertainty of the model and to reduce the overfitting of the model (Fig. 6b). We used a probability of 0.3 in both layers. The original DenRes-131^28^ combines four blocks from ResNet-50 and DenseNet-121 with width, height, and frames of $58 \times 58 \times 256$, $28 \times 28 \times 512$, $14 \times 14 \times 1024$, and $7 \times 7 \times 2048$, respectively. Each of the four outputs feeds a block of convolution and average pooling layers. The final layer uses a soft-max regression, so that the network can conclude in the classification decision (Fig. 1b). For all the networks we used a three level multi-preceptor tuner layer and a combination of two Monte Carlo dropout layers to estimate the epistemic uncertainty of the model. We utilized the trained weights of the networks to classify the patches in one of the six classes (Fig. 1c). After we used these annotated patches to rebuild back the 3D anatomical model of the lungs. We defined the portions of each of the six diseased classes and we extracted the pulmonary hypertension profile of the specific patient.

#### Police learning

After random shuffling each dataset had been partitioned into $70\%$ and $30\%$ of the total number of images to train and validate the networks. We used categorical cross-entropy as a cost function. The loss function was optimized using the stochastic gradient descent (SGD) method with a fixed learning rate of 0.0001. We applied transfer learning techniques to the networks using the ImageNet dataset (http://www.image-net.org). The ImageNet dataset consists of over 14 million images and the task were to classify the images into one of almost 22, 000 different categories (cat, sailboat, etc.). We trained the DenRes-131 for 25 epochs and the other three networks for 100 epochs.

### Explainability and uncertainty estimation

The explainable analysis and uncertainty estimation of a network is a very important part of a classification study as it validates the functional, operational, safety and usability dimensions of a transparent AI tool. In this study we used an established local explainable technique in medical imaging applications, the GRAD-CAM method^32^. However the use of only local explainability techniques can be biased. Thus we tried to remove the bias effect by using an interpretable non-linear dimensionality reduction technique, the principal component analysis (PCA). We utilized the PCA to study the variability and generalization of all the GradCam outputs (PCA-GradCam) and all the input patch images (PCA-Shape) of the testing cohort to evaluate the learning pattern of the deep learning network. We tested three different values of components for the PCA analysis (4, 8 and 16), and we studied their differences. We extracted the correlation coefficient of the PCA-GradCam and PCA-Shape to compare the positive and negative ratio between them. Therefore, we evaluated the similarities, and the accuracy in correct and wrong learning patterns of the networks.

The uncertainty of our multi-classification task was separated into aleatoric and epistemic uncertainty. Aleatoric uncertainty captures noise inherent during the data collection. This noise can be product of different reasons such as variation of biological (age, immunity level, gender, biochemical parameters) and environmental (lifestyle, emotional state, anxiety, stress, climate) conditions, social status (family support, friends’ interaction, financial security etc.) or variability in the scanning machines or medical tools were used for the medical data collection. On the other hand, epistemic uncertainty studies the uncertainty in the model’s prediction based on the variability of the network’s parameters^33^. Epistemic uncertainty refers mainly to lack of knowledge of the way to solve a specific medical problem (features and parameters involved in study). For example, when someone develops a machine learning network to solve a cancer risk assessment problem, he takes into consideration specific biomarkers (features) related with the prediction of the severity level of each sample. Because there is a gap of knowledge of other possible biomarkers which can contribute to the risk assessments of each sample, he needs to include the possibility that other networks with different parameters can solve the same problem. Monte Carlo dropout method samples the training data for limited iterations and it generates an estimation of the posterior distribution (network with trained parameters). Thus, we estimated the posterior distribution providing information on whether the input data exists in the learned distribution. MC dropout is the most common way to estimate the epistemic uncertainty in Bayesian networks^33^. To estimate the aleatoric uncertainty we used a Monte Carlo test-time augmentation method^34^. The uncertainty can be estimated by using the variance or the entropy distribution $p(Y \mid X)$. Here we utilized the entropy distribution given by:

1 $$\begin{aligned} H(Y \mid X)= - \int { p(y \mid X) ln(p(y \mid X))} dy \end{aligned}$$

We used a Monte Carlo simulation of $n=21$ samples of data augmented patches (rotation, shrink, scale, noise) to extract prediction results of $Y={y_1,y_2,\ldots y_N}$. Suppose there are M unique values in Y. For classification tasks, this typically refers to M labels. Assume the frequency of the *m* unique value is $p_m$, then $H(Y \mid X)$ is approximated as:

2 $$\begin{aligned} H(Y \mid X)= - \sum _{m=1}^{M} p_m ln(p_m) \end{aligned}$$

The frequency of the prediction of a specific patch is given by $p_m=arg_{y}max(p(y \mid X))$.

The epistemic uncertainty was estimated by using Monte Carlo dropout layers in the multi-preceptor level of the networks (Fig. 1b). The simulation number was again T = 21 (where T the times that the dataset feds to the networks). The average result was given by:

3 $$\begin{aligned} E(y)= \frac{1}{T} \sum _{t=1}^T{x_t} \end{aligned}$$

The epistemic uncertainty computed by the variance operation of:

4 $$\begin{aligned} Var(y)= \sigma ^2 + \frac{1}{T} \sum _{t=1}^T{f(y_t(x))^T f(y_t(x)) - E[y]^T E[y]} \end{aligned}$$

where *x* denotes the input features from training images, the predictive mean *E*[*y*] denotes the expected model output given the input *x*, and $\sigma ^2$ denotes the aleatoric uncertainty. This process was repeated *T* times for *T* independent identical distributions ${y_1(x),\ldots ,y_T(x)}$. These output values are empirical samples from an approximate predictive distribution.

### Statistical analysis

Continuous variables were presented as proportions, means ± standard deviations, or median and interquartile range for data not following a normal distribution. We used an ANOVA analysis with $\text {{p}}$-value 0.05 to calculate the statistically significantly differences between the different deep learning classifiers. The statistical analyses were carried out using the lifelines and Python^35^) and R libraries^36^.

### Ethics approval

The methods were performed in accordance with relevant guidelines and regulations and approved by ASPIRE registry (Assessing the Spectrum of Pulmonary Hypertension Identified at a Referral Centre), reference c06/Q2308/8; REC 17/YH/0016.

## Supplementary Information

Supplementary Information.

## Data Availability

This study has the appropriate research ethics committee approval of ASPIRE registry (Assessing the Spectrum of Pulmonary Hypertension Identified at a Referral Centre), reference c06/Q2308/8; REC 17/YH/0016. The data are available as requested from the corresponding author Dr. Andy Swift.

## Code availability

The code developed in this study is written in the Python programming language using Keras/TensorFlow (Python) libraries. For training and testing of deep learning networks, we have used an NVIDIA cluster (JADE2) with 4 GPUs and 64 GB RAM memory. The code is publicly available in https://github.com/INSIGNEO/PH_3Dpatches.
