# Supplementary material for: A transparent artificial intelligence framework to assess lung disease in pulmonary hypertension
Source: Sci Rep. 2023 Mar 7;13:3812. doi: 10.1038/s41598-023-30503-4 (PMC9990015; doi:10.1038/s41598-023-30503-4)
Supplement: Supplementary file 1 — Supplementary Information. [file 41598_2023_30503_MOESM1_ESM.pdf]

A transparent artificial intelligence framework to assess lung disease in pulmonary hypertension: Supplementary material

Michail Mamalakis[1,2,4*], Krit Dwivedi[1,4], Michael Sharkey[1], Samer Alabed[1,4], David Kiely[1,3] and Andrew J. Swift[1,4*]

[1*]Department of Infection, Immunity & Cardiovascular Disease, University of Sheffield, Beech Hill Rd, Sheffield, S10 2RX, UK.
[2]Department of Computer Science, University of Sheffield, 211 Portobello, Sheffield, S1 4DP, UK.
[3]Department of Cardiology, University of Sheffield, Sheffield Teaching Hospitals Sheffield , Sheffield, S5 7AU, UK.
[4]Insigneo Institute for in silico Medicine, University of Sheffield, The Pam Liversidge Building, Sheffield, S1 3JD, UK.

*Corresponding author(s). E-mail(s): m.mamalakis@sheffield.ac.uk; a.j.swift@sheffield.ac.uk;
Contributing authors: kdwivedi1@sheffield.ac.uk; m.j.sharkey@sheffield.ac.uk; s.alabed@sheffield.ac.uk; d.g.kiely@sheffield.ac.uk;

Abstract

Recent studies have recognized the importance of characterizing the extent of lung disease in pulmonary hypertension patients by using Computed Tomography as the routine scanning protocol. Even though texture extraction and deep learning feature classification are rapidly growing fields of medical artificial intelligence research, there is a lack of application in pulmonary hypertension. The trustworthiness of an artificial intelligence system is linked with the depth of the evaluation in functional, operational, usability, safety and validation dimensions. The safety and validation of an artificial tool is linked to the uncertainty estimation of the model's prediction. On the other hand, the functionality, operation and usability can be achieved by explainable deep learning

approaches which can verify the learning patterns and use of the network from a generalized point of view. We developed an artificial intelligence framework to map the 3D anatomical models of patients with evidence of pulmonary hypertension. To this end, we studied a patched oriented multi-classification task of pulmonary hypertension by using different deep learning networks. To verify the trustworthiness of the framework we studied the uncertainty estimation of the network's prediction, and we explained the learning patterns of the network. Therefore a new generalized technique combines local explainable and interpretable dimensionality reduction approaches (PCA-GradCam, PCA-Shape) was developed. Our framework was evaluated in an unbiased validation scheme with 'seen' and 'unseen' datasets achieving accurate, robust and generalized results in the 'unseen' dataset (91.83 $\pm$ 3.48 % Jaccard score, 5.96 $\pm$ 3.17 mm Hamming distances, 80.21 $\pm$ 7.83 % MCC, 93.69 $\pm$ 3.90 accuracy, and 0.855 $\pm$ 0.40 Root Mean Square Error).

Keywords: DenRes-131, Classification, Pulmonary Hypertension, 3D patches, Explainable AI, Uncertainty

1 Overview

In this document we extend the study of *A trustworthy artificial intelligence framework to assess lung disease in pulmonary hypertension* with some extra information related to methodology implementation, and result (Tables and Figures). The structure we follow is; Section 2 Methodology development, and Section 3 Results.

2 Methodology development

We developed a patch-oriented AI framework to map the 3D anatomical models of patients with lung disease in pulmonary hypertension. The framework has four steps: the extraction of the 3D patches, the classification of each patch, the volume rebuild of the 3D anatomical model, and the analysis and evaluation of the pulmonary hypertension profile of the lungs (S S1 manuscript).

2.1 Patch sizes parameters initialization

For the extraction of different patch sizes we used a variation of width, height and depth sizes (64x64x3, 32x32x3, 16x16x8, 8x8x3). To verify that each patch will include sufficient amount of lung information, we utilised a ratio about the lung segmented area which at least needed to be included in each patch. This ratio was at 80 %. We used a sliding windows to extract the patches of each patient without any overlapping of the windows.

2.2 Multi-classifiers

During the training we did not use any cross-validation protocol as the cohorts size was very large (over 5000 patches per class). Therefore we utilized a validation split protocol of 70 % training and 30 % validating samples to avoid any overfitting or biased effect of the training process.

2.3 Datasets and cohorts

The training validation and testing datasets (internal and unseen cohorts) can be provided after submission request to Senior author or correspond author.

2.4 Explanation of network's learning patterns of the generalized technique

Two experts AS and KD were visually inspected the results of Figure 6a,6c and Figure 7. In Figure 7 in each class, was PCA-Shape and PCA-GradCam images for each PCA component (first, second etc.). The PCA-Shape was the PCA of the input patches of each class region. In most of the cases in the PCA-Shape images the blue colour was correspond to lungs and the green to other 'no interest' regions (other organs, bones etc.). For the PCA-GradCam images the colour scale was from blue to red colour (red high focused pixels blue no focused). The correlation coefficient of the two images gives positive (blue colour) and negative correlation pixels (red colour) between the PCA-GradCam and PCA-Shape images. The negative correlation pixels meant that in these pixels the intensity level of the two images was inverse. For example, if the PCA-GradCam pixels had red colour then the PCA-Shape pixels had blue colour. In the positive correlation the two images had the same intensity orientation (both red scale). If the pixels correlation was 1 or -1 meant that the images had the same corresponding level of intensity either same orientation or inverse (the high or low intensity). If the correlation was smaller than 1 or -1 meant that the intensity correlation was weak, thus the two images had different level of pixels' intensity. When we computed the negative ratio and positive ratio, we computed the average number of positive and negative pixels' correlation in each case. If the one ratio was higher than the other meant that the network in that case focused on the inverse (negative) or on the same (positive) orientation as in the PCA-Shape image. As higher as the differences were between the positive and negative ratios, the stronger the positive or negative focusing of the network was. Regarding the correct or wrong learning pattern, the experts' needed to check the PCA-Shape images and observed in which area the model needed to focus. They needed to determine if it was the blue or green colour areas of the PCA-Shape image. After, we evaluated the results of the PCA-GradCam images and based on the above terminology we evaluated the positive and negative ratio. Finally, we did the same methodology to all the images of Fig 7. and we determined the correct or wrong learning patterns of the network.

Fig. S1: Train and validation results of different metrics and patch sizes.

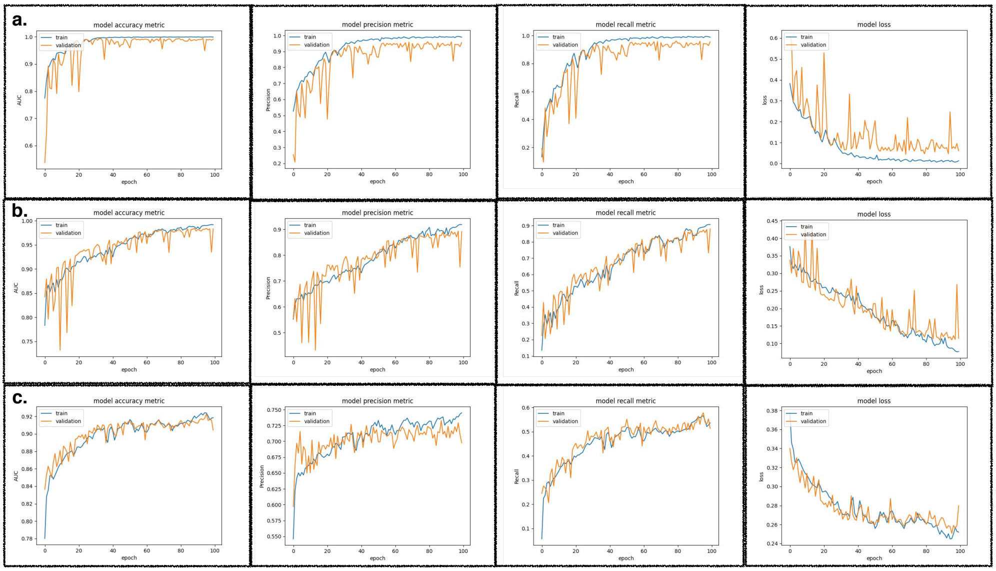

The patch sizes from **a-c** are 64x64x3, 32x32x3, and 16x16x3 respectively. The plots verify that the networks during training avoid any possible overfitting. This figures summarize the results of DenseNet-121 network. The validation signal is the orange lines and the training signal with the blue lines.

3 Results

Figure S1 shows the training and validation results of different metrics and patch sizes (64,32,16 a-c). These plots verify that the networks did not overfitted as the validation (orange lines) and training (blue lines) signals are similar. This figures summarizes the results of DenseNet-121 network. Tables S1, S2 S3, and S4 summarize the results of all the networks (Vgg-16, ResNet-50,DenseNet-121 and DenRes-131) in the validating and testing 'seen' cohorts of the multi-classification task. We extracted the manuscript's Figure S1 based on the Tables S1, S2 S3, and S4 scores. The AUC-ROC curves, precision, recall, and f1-score metrics have been used to evaluate the generalization and accuracy of the networks' classification. Fig. S2 presents the AUC-ROC curves of different deep learning models (DenseNet-121 and DenRes-131) on the datasets.

Table S1: Quantitative evaluation metrics for the test case on the 'seen' pulmonary hypertension Sheffield dataset.

64X64X3 patch size				
Metric (%)	Vgg16	Resnet50	DenseNet121	DenRes-131
Recall	18.23	20.23	86.26	82.01
Precision	91.44	80.93	86.46	82.25
AUC-ROC sample	54.86	55.35	91.88	90.12
AUC-ROC macro	53.98	54.87	86.59	90.01
AUC-ROC micro	54.86	55.35	91.88	90.00
AUC-ROC weighted	54.00	54.69	90.14	87.75
F1 sample	30.48	32.37	86.46	82.10
F1 macro	23.31	22.38	78.55	82.81
F1 micro	30.48	32.37	86.46	82.00
F1 weighted	28.01	33.58	86.56	82.33
32X32X3 patch size				
Metric (%)	Vgg16	Resnet50	DenseNet121	DenRes-131
Recall	18.41	35.72	74.05	81.49
Precision	92.15	71.45	74.23	81.67
AUC-ROC sample	55.29	64.29	84.44	88.91
AUC-ROC macro	54.23	62.39	80.14	83.94
AUC-ROC micro	55.19	64.28	84.44	88.86
AUC-ROC weighted	54.78	63.29	83.87	88.38
F1 sample	30.71	47.63	74.01	81.55
F1 macro	23.63	17.31	45.21	66.85
F1 micro	30.71	47.63	74.04	81.53
F1 weighted	28.43	51.94	79.05	83.27
16X16X3 patch size				
Metric (%)	Vgg16	Resnet50	DenseNet121	DenRes-131
Recall	18.74	60.00	62.14	61.12
Precision	93.72	60.01	62.15	61.20
AUC-ROC sample	56.23	75.96	77.28	76.58
AUC-ROC macro	55.66	76.78	78.84	77.31
AUC-ROC micro	56.22	75.95	77.28	76.34
AUC-ROC weighted	55.43	75.40	76.98	76.01
F1 sample	31.24	60.00	62.15	61.12
F1 macro	26.08	57.64	51.82	53.48
F1 micro	31.24	59.93	62.15	60.87
F1 weighted	31.30	60.11	64.76	63.79

Fig. S2: The ROC curves results of DenseNet-121, and DenRes-131 (DENRES) for the multi-classification task.

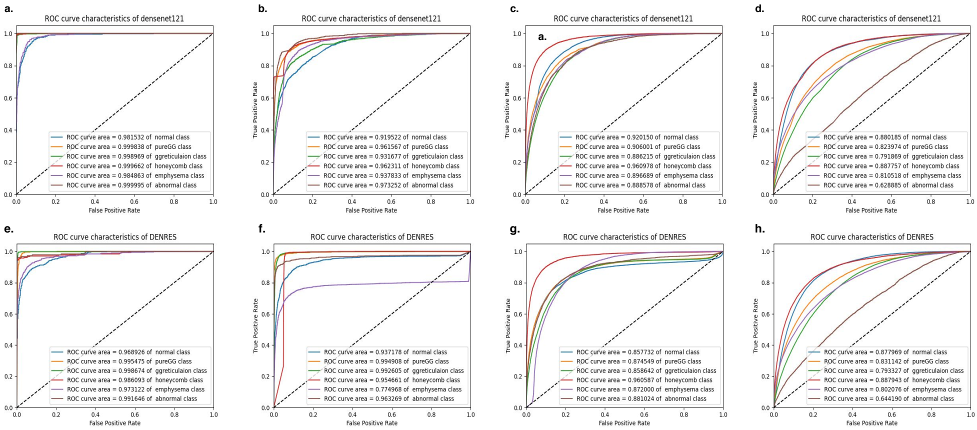

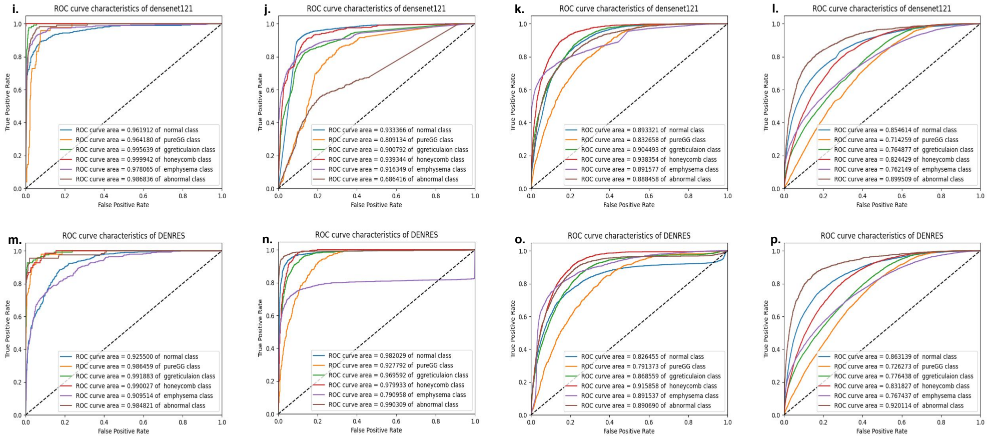

a-h the ROC curve result of a variate of volume patches in the 'seen' validation cohort. **i-p** the ROC curve result of a variate of volume patches in the 'seen' testing cohort. From left to right a-d, e-h, k-n and o-r the different 3D patch sizes $64 \times 64 \times 3$, $32 \times 32 \times 3$, $16 \times 16 \times 3$, and $8 \times 8 \times 3$ height, width and depth respectively. The networks are presented are the DenseNet-121 and DenRes-131.

Table S2: Quantitative evaluation metrics for the test case on the 'seen' pulmonary hypertension Sheffield dataset.

8X8X3 patch size				
Metric (%)	Vgg16	Resnet50	DenseNet121	DenRes-131
Recall	19.34	21.81	48.07	50.60
Precision	92.13	87.51	48.17	50.62
AUC-ROC sample	55.67	57.81	68.84	70.17
AUC-ROC macro	54.76	56.98	67.33	67.27
AUC-ROC micro	57.63	58.01	68.56	70.01
AUC-ROC weighted	56.43	57.21	67.66	68.65
F1 sample	34.54	35.00	48.07	50.11
F1 macro	22.33	23.87	36.50	42.13
F1 micro	36.98	35.00	48.07	49.60
F1 weighted	35.43	35.81	51.09	52.68

Table S3: Quantitative evaluation metrics of 70/30 validation split on the 'seen' pulmonary hypertension Sheffield dataset.

64X64X3 patch size				
Metric (%)	Vgg16	Resnet50	DenseNet121	DenRes-131
Recall	20.00	18.65	92.50	91.89
Precision	99.00	93.29	92.58	91.91
AUC-ROC sample	51.12	55.97	95.51	94.89
AUC-ROC macro	50.78	54.66	95.29	94.63
AUC-ROC micro	51.00	56.01	95.47	94.81
AUC-ROC weighted	51.08	54.67	95.37	94.78
F1 sample	28.57	31.09	92.53	91.15
F1 macro	28.15	26.12	92.81	91.32
F1 micro	28.57	31.09	92.51	91.17
F1 weighted	28.15	31.34	95.37	91.32
32X32X3 patch size				
Metric (%)	Vgg16	Resnet50	DenseNet121	DenRes-131
Recall	16.66	18.66	74.33	85.27
Precision	99.00	93.29	74.73	85.41
AUC-ROC sample	46.79	55.97	84.63	91.18
AUC-ROC macro	45.77	55.47	86.09	90.74
AUC-ROC micro	45.99	55.98	84.52	91.12
AUC-ROC weighted	46.02	55.21	84.33	91.31
F1 sample	28.57	31.09	74.46	85.32
F1 macro	28.15	26.11	74.58	84.20
F1 micro	28.57	31.09	84.52	85.29
F1 weighted	28.15	31.34	73.78	85.78
16X16X3 patch size				
Metric (%)	Vgg16	Resnet50	DenseNet121	DenRes-131
Recall	18.84	61.23	64.69	64.82
Precision	94.19	61.31	64.84	65.01
AUC-ROC sample	56.51	76.78	78.83	78.92
AUC-ROC macro	56.01	76.74	78.42	78.68
AUC-ROC micro	56.51	76.77	78.80	78.41
AUC-ROC weighted	56.00	76.35	78.44	78.47
F1 sample	31.39	61.30	64.74	64.81
F1 macro	26.33	59.75	61.09	61.63
F1 micro	31.39	61.31	64.74	64.88
F1 weighted	31.60	61.44	65.27	64.43

Table S4: Quantitative evaluation metrics of 70/30 validation split on the 'seen' pulmonary hypertension Sheffield dataset.

8X8X3 patch size				
Metric (%)	Vgg16	Resnet50	DenseNet121	DenRes-131
Recall	20.92	21.3	53.66	53.71
Precision	83.72	83.71	53.65	53.78
AUC-ROC sample	55.32	56.39	72.19	72.80
AUC-ROC macro	54.12	54.21	68.93	69.05
AUC-ROC micro	56.24	56.40	72.19	72.83
AUC-ROC weighted	55.43	55.76	71.46	72.32
F1 sample	33.49	33.49	53.65	54.01
F1 macro	22.97	23.01	45.46	46.32
F1 micro	33.48	33.49	53.65	54.67
F1 weighted	34.45	34.46	55.06	55.87
